# Resistance profiles, virulence and antimicrobial resistance genes of XDR *S.* Enteritidis and *S.* Typhimurium

**DOI:** 10.1186/s13568-023-01615-x

**Published:** 2023-10-10

**Authors:** Abdelazeem M. Algammal, Reham M. El-Tarabili, Wafaa A. Abd El-Ghany, Enas A. Almanzalawi, Tahani M. Alqahtani, Hanaa Ghabban, Amenah S. Al-otaibi, Nayera M. Alatfeehy, Naira M. Abosleima, Helal F. Hetta, Ghada A. Badawy

**Affiliations:** 1 Department of Bacteriology, Immunology, and Mycology, Faculty of Veterinary Medicine, Suez Canal University, Ismailia, 41522 Egypt; 2 Poultry Diseases Department, Faculty of Veterinary Medicine, Cairo University, Giza, 12211 Egypt; 3 Department of Biological Sciences, Faculty of Sciences, King Abdulaziz University, 21589 Jeddah, Saudi Arabia; 4 Department of Biology, Faculty of Science, University of Tabuk, 71491 Tabuk, Saudi Arabia; 5 Reference Laboratory for Veterinary Quality Control on Poultry Production, Animal Health Research Institute, Agriculture Research Center, Dokki, Giza, 1261 Egypt; 6 Department of Medical Microbiology and Immunology, Faculty of Medicine, Assiut University, Assiut, 71515 Egypt; 7 Botany Department, Faculty of Science, Fayoum University, Fayoum, 63514 Egypt; 8 Department of Biology, Faculty of Science, University of Tabuk, 46429 Umluj, Saudi Arabia

**Keywords:** *S*. Typhimurium, *S.* Enteritidis, XDR, Diseased broilers, Virulence genes, Antibiotic resistance genes

## Abstract

Avian salmonellosis is concomitant with high financial crises in the poultry industry as well as food-borne illness in man. The present study is designed to investigate the emergence of *Salmonella* Enteritidis and *Salmonella* Typhimurium in diseased broilers, resistance profiles, and monitoring virulence and antibiotic resistance genes. Consequently, 450 samples (cloacal swabs, liver, and spleen) were collected from 150 diseased birds from different farms in Giza Governorate, Egypt. Subsequently, the bacteriological examination was done. Afterward, the obtained *Salmonella* isolates were tested for serogrouping, antibiogram, PCR monitoring of virulence (*inv*A, *stn,* *hil*A, and *pef*A), and antimicrobial resistance genes (*bla*_TEM_, *bla*_CTX-M_, *bla*_NDM_, *erm*A, *sul*1, *tet*A, and *aad*A1). The total prevalence of *Salmonella* in the examined diseased broilers was 9.3%, and the highest prevalence was noticed in cloacal swabs. Among the recovered *Salmonella* isolates (*n* = 35), 20 serovars were recognized as *S*. Enteritidis and 15 serovars were identified as *S*. Typhimurium. Almost 60% of the retrieved *S.* Enteritidis serovars were extensively drug-resistant (XDR) to seven antimicrobial classes and inherited *sul*1, *bla*_TEM_, *tet*A*,* *bla*_CTX-M_, *ere*A, and *aad*A1 genes. Likewise, 25% of the recovered *S.* Enteritidis serovars were multidrug-resistant (MDR) to six classes and have *sul*1, *bla*_TEM_, *tet*A*,* *bla*_CTX-M_, and *ere*A resistance genes. Also, 66.7% of the retrieved *S.* Typhimurium serovars were XDR to seven classes and have *sul*1, *bla*_TEM_, *tet*A*,* *bla*_CTX-M_, *ere*A, and *aad*A1 genes. Succinctly, this report underlined the reemergence of XDR *S*. Typhimurium and *S.* Enteritidis in broiler chickens. Meropenem and norfloxacin exposed a hopeful antimicrobial activity toward the re-emerging XDR *S*. Typhimurium and *S.* Enteritidis in broilers. Moreover, the recurrence of these XDR *Salmonella* strains poses a potential public health threat.

## Introduction

Salmonellosis is a serious zoonotic disease that has significant public health importance. Non-typhoidal *Salmonellae* are imperative foodborne pathogens associated with the digestive tract of animals and birds (Hunter and Watkins 2018). *Salmonella* is a ubiquitous pathogen that causes clinical or subclinical infection in asymptomatic birds, known as carriers. Globally, non-typhoidal *Salmonella* is incriminated in more than 150 million reports of gastroenteritis as well as about 56,000 mortalities every year (Mezal et al. 2014). *Salmonella* infection results in tremendous financial losses in the poultry industries due to treatment costs, and poor growth, and high mortalities. Moreover, it causes food poisoning in man (Shafiullah et al. 2016).

*Salmonella* is a Gram-negative, facultative anaerobic bacterium belonging to the family *Enterobacteriaceae*. Most *Salmonella* serovars are motile except *S.* Pullorum and *S*. Gallinarium (Shafiullah et al. 2016; Zhao et al. 2017). *S.* Typhimurium and *S*. Enteritidis are the utmost predominant non-typhoidal *Salmonella* species that incriminated in gastroenteritis in both humans and animals. The infection is commonly associated with diarrhea, fever, vomiting, and severe abdominal pain 12–36 h after ingestion of the contaminated food (Mezal et al. 2013; Zhao et al. 2020). Salmonellosis in poultry leads to prolonged fecal shedding and severe infection in hens and chicks. In experimentally infected birds with *S*. Enteritidis, disparities in mortalities, severity of infection, rate of production of contaminated eggs, and fecal shedding were noticed. The severity of infection is affected by the inoculum size, *Salmonella* serovar, and bird age (Kumar et al. 2019; Li et al. 2020).

*Salmonella* is an optional intracellular pathogen. The pathogenicity of *Salmonella* is governed by various determinants, which are regulated by its capability to attach to the host cells, invade different cells, intracellular survival, and multiplication in the host enterocytes (Eng et al. 2016). The main virulence determinants that exert a vital role in *Salmonella* pathogenicity include adhesion and invasion to the target cells, intracellular survival and growth, iron acquirement, and toxin production. Several virulence-determinant genes are assembled together in definite genomic elements called *Salmonella* Pathogenicity Islands (SPIs), gained by genetic transfer between bacterial pathogens (Tamang et al. 2014; Litrup et al. 2010). The *inv*A gene is the most prominent virulence gene of *Salmonella* that exerts a remarkable role in host invasion. Moreover, the *inv*A gene is conserved in different *Salmonella* species. Furthermore, other genes such as *stn* (*Salmonella*-enterotoxin), *hil*A, and *pef*A (the plasmid-encoded fimbriae) are the key virulence determinant genes associated with salmonellosis (Gole et al. 2013; Webber et al. 2019).

In the last decade, multidrug resistance has conspicuously augmented globally, which indicates a public health risk. Various reports highlighted the existence of multidrug-resistant (MDR), extensively drug-resistant (XDR), and pan drug-resistant (PDR) bacterial strains (superbugs) from distinctive sources, such as animals, birds, humans, fish, and different food products (Algammal et al. 2021, 2022; Hetta et al. 2021; Elbehiry et al. 2022; Kozytska et al. 2023). The MDR patterns of *Salmonella* serovars to different antimicrobial classes (especially sulfonamides, aminoglycosides, tetracyclines, and penicillins) formerly recorded by several investigations (Shafiullah et al. 2016; Alam et al. 2020; Zaho et al. 2020).

The present study directed to determine the prevalence of *S.* Enteritidis and *S.* Typhimurium in diseased broilers, antimicrobial susceptibility testing, and PCR-based screening of virulence (*inv*A, *stn,* *hil*A, and *pef*A) and antibiotic resistance genes (*bla*_TEM_, *bla*_CTX-M_, *bla*_NDM_, *erm*A, *sul*1, *tet*A, and *aad*A1) in the recovered *Salmonella* serovars.

## Materials and methods

### Sampling

Approximately 450 samples (cloacal swabs, liver, and spleen; *n* = 150 for each) were collected from 150 diseased broilers (4–6 weeks old age) from commercial farms in Giza Governorate, Egypt (from April to May 2021). The examined diseased broilers suffered from diarrhea, depression, and reduced growth performance. Post-mortem examination of sacrificed and recently dead chickens revealed dehydration, enlarged congested liver, and enlarged spleen. Moreover, the postmortem findings were uniform in most of the examined birds. Samples were obtained aseptically, placed in an ice box, and conveyed to the laboratory immediately for bacteriological examination.

### Isolation and identification of *Salmonella*

The obtained samples (1 g of each liver and spleen sample) were inoculated in 9 ml buffered peptone water (BD Difco, Thermo Fisher Scientific, Waltham, USA) and incubated at 37 °C for 18 h. Afterward, 0.1 ml of the incubated broth was inoculated in 10 ml of Rappaport–Vassiliadis broth (BD Difco, Thermo Fisher Scientific, Waltham, USA), a selective enrichment medium, and left incubated at 42 °C for 18 h. Then a loopful from the incubated broth was streaked on Xylose Lysine Deoxycholate agar (XLD), Hektoen Enteric Agar (HEA), and MacConkey agar (BD Difco, Thermo Fisher Scientific, Waltham, USA) plates and left incubated for 24 h. at 37 °C (ISO 6579–1 2017; Abd El-Aziz et al. 2021). The identification of *Salmonella* was performed consistent with Gram’s staining, cultural features, and the biochemical reactions (oxidase, Voges-Proskauer, catalase, H_2_S production, methyl red, nitrate reduction, sugar fermentation tests, indole production, citrate utilization, and urease test) according to Quinn et al. (2002). Besides, the identification of *Salmonella* was ensured genetically by the PCR amplification of the *inv*A gene (Oliveira et al. 2003).

### *Serologicaltyping*

The retrieved *Salmonella* isolates were subjected to serological identification consistent with (Kauffmann and Das Kauffmann 2001) using diagnostic polyvalent and monovalent *Salmonella* “O” and “H” antisera (Sifin Diagnostics, Gmbh, Berlin, Germany).

### Antibiogram of the retrieved *Salmonella* serovars

The obtained serovars were tested for susceptibility to 11 antibiotic discs using the disc diffusion method on Muller-Hinton agar (Difco, USA). The test was applied according to the guidelines of CLSI (2018). The following discs were used, norfloxacin (NOR, 10 μg), gentamycin (GEN, 10 μg), meropenem (MEM, 10 μg), erythromycin (E, 15 μg), amoxicillin (AM, 30 μg), ceftazidime (CAZ, 30 μg), sulfamethoxazole–trimethoprim (SXT, 30 μg), amoxicillin–clavulanic acid (AMC, 30 μg), oxytetracycline (OX, 30 μg), neomycin (NEO, 10 μg), and cefotaxime (CTX, 30 μg) (Oxoid, UK). Likewise, *E.* *coli*-ATCC 25922 was used as a control strain. The retrieved *Salmonella* serovars were classified as multidrug-resistant (MDR) and extensively drug-resistant (XDR), consistent with (Magiorakos et al. 2012). Furthermore, the multiple antibiotic resistance (MAR) index (the ratio between the number of antimicrobial agents that one strain is resistant to and the total number of tested antimicrobial agents) was estimated consistent with Krumperman (1983).

### PCR monitoring of virulence determinant and resistance genes in the obtained *Salmonella* serovars

PCR was used to monitor the distribution of the virulence (*inv*A, *hil*A, *stn*, and *pef*A genes) and resistance genes (*bla*_TEM_, *bla*_CTX-M_, *bla*_NDM_, *erm*A, *sul*1, *aad*A1, and *tet*A) among the recovered *Salmonella* serovars. The *g*DNA of the tested *Salmonella* serovars was extracted using a genomic DNA extraction Kit (Invitrogen, Carlsbad, USA). Moreover, positive (positive strains obtained from the A.H.R.I, Egypt) and negative controls (reactions with DNA-free reactions); were used. The used primers (Thermo Fisher Scientific, Karlsruhe, Germany) and PCR protocols were clarified in Table 1. The amplified PCR products were screened by the agar gel electrophoresis (1.5% agarose stained with 10 mg/ml ethidium bromide). Afterward, the gel was photographed.

**Table 1:** List of used primers used in this study

Target	Genes	Primers sequences	Product size (bp)	PCR thermal profile (35 cycles)			References
				Denaturation	Annealing	Extension	
Virulence genes	*inv*A	F:TCATCGCACCGTCAAAGGAACC R:GTGAAATTATCGCCACGTTCGGGCAA	284	95 °C 30 s	55 °C 30 s	72 °C 90 s	Oliveira et al. (2003)
	*hil*A	F:CGGAAGCTTATTTGCGCCATGCTGAGGTAG R:GCATGGATCCCCGCCGGCAGAGTTGTG	854	95 °C 30 s	55 °C 30 s	72 °C 90 s	Cardona-Castro et al. (2002)
	*pef*A	F: TGTTTCCGGGCTTGTGCT R: CAGGGCATTTGCTGATTCTTCC	700	95 °C 30 s	55 °C 30 s	72 °C 90 s	Murugkar et al. (2003)
	*stn*	F:TTGTGTCGCTATCACTGGCAACC R: ATTCGTAACCCGCTCTCGTCC	617	95 °C 30 s	55 °C 30 s	72 °C 90 s	
Resistance genes	*tet*A	F:GGTTCACTCGAACGACGTCA R:CTGTCCGACAAGTTGCATGA	576	94 °C 30 s	55 °C 40 s	72 °C 45 s	Randall et al. (2004)
	*sul*1	F:CGGCGTGGGCTACCTGAACG R:GCCGATCGCGTGAAGTTCCG	433	94 °C 30 s	54 °C 40 s	72 °C 45 s	Ibekwe et al. (2011)
	*aad*A1	F: TATCAGAGGTAGTTGGCGTCAT R:GTTCCATAGCGTTAAGGTTTCATT	484	94 °C 30 s	50–54 °C 40 s	72 °C 45 s	Randall et al. (2004)
	*ere*A	F: GCCGGTGCTCATGAACTTGAG R: CGACTCTATTCGATCAGAGGC	419	94 °C 30 s	58 °C 30 s	72 °C 60 s	Van et al. (2008)
	*bla*_CTX-M_	F:ATG TGC AGY ACC AGT AAR GTK ATG GC R:TGG GTR AAR TAR GTS ACC AGA AYC AGC GG	593	94 °C 30 s	54 °C 40 s	72 °C 45 s	Archambault et al. (2006)
	*bla*_TEM_	F:ATCAGCAATAAACCAGC R:CCCCGAAGAACGTTTTC	516	94 °C 30 s	54 °C 40 s	72 °C 45 s	Colom et al. (2003)
	*bla*_NDM_	F:GGCGGAATGGCTCATCACGA R:CGCAACACAGCCTGACTTTC	287	94 °C 30 s	55 °C 40 s	72˚C 30 s	Xia et al. (2012)

### *Statisticalanalysis*

The obtained data were analyzed using the Chi-square test (SAS software, 9.4 M6, SAS Institute, Cary, NC, USA), whereas a *p*-value < 0.05 points to a significant difference between the obtained data*.* The findings of the antibiogram were illustrated by a heatmap using GraphPad Software (version 8.0.1, GraphPad Software Inc., La Jolla, CA, USA). A heatmap with hierarchical clustering was accomplished to illustrate the occurrence of the antimicrobial resistance phenotypes and antimicrobial resistance genes in the retrieved serovars using the “Pheatmap” package in R software (version 4.0.2; https://www.r-project.org/). Also, the R-software was used to estimate the correlation coefficient between phenotypic resistance patterns and resistance genes. Moreover, the association between different variables was performed.

## Results

### Phenotypic traits of the retrieved *Salmonella* serovars

The retrieved *Salmonella* colonies were transparent with a black center on Hektoen Enteric agar, red colonies with a black center on XLD, and small pale (non-lactose fermenter) smooth, transparent colonies on MacConkey agar. Moreover, the microscopical examination revealed Gram-negative, non-spore-forming rods. Furthermore, the obtained *Salmonella* serovars tested positive for citrate utilization, catalase, methyl red, H_2_S production, and nitrate reduction tests. In contrast, the isolated *Salmonella* serovars tested negative for oxidase, urease, Voges–Proskauer, and indole tests.

### Prevalence of *Salmonella* serovars in the examined diseased birds

**Fig. 1:**
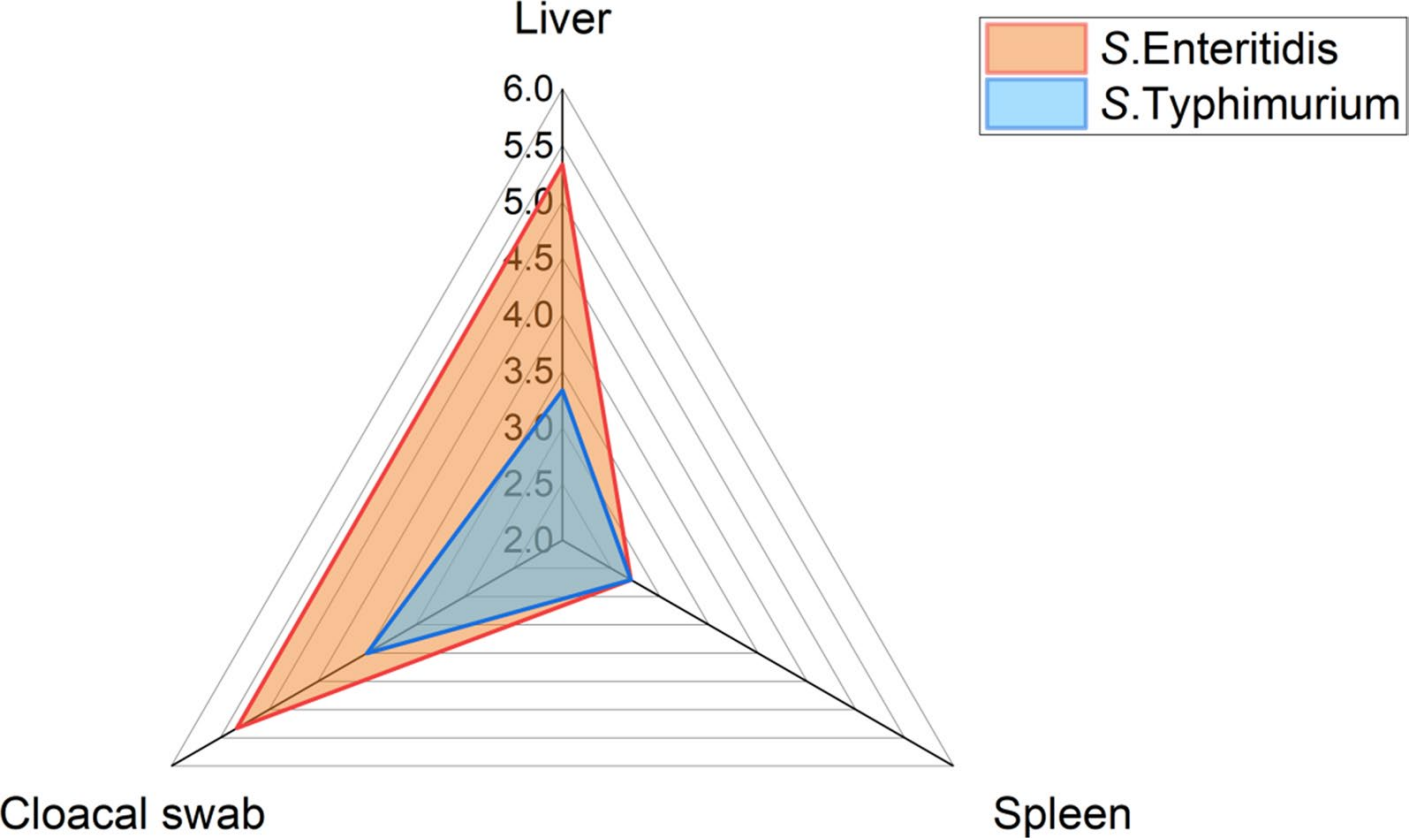
Prevalence of *Salmonella* serovars between various examined samples collected from diseased broilers

Herein, the total prevalence of *Salmonella* in the examined diseased broilers was 9.3% (14/150). In the present study, 35 (7.8%) *Salmonella* isolates (20 *S*. Enteritidis and 15 *S*. Typhimurium) were isolated from 450 bacteriologically examined samples collected from 150 diseased birds. The prevalence of *S*. Enteritidis was 5.3%, 5.3%, and 2.7% in the examined cloacal swabs, liver, and spleen samples. Moreover, the prevalence of *S*. Typhimurium was 4%, 3.3%, and 2.7% in the examined cloacal swabs, liver, and spleen samples. There is no significant difference in the distribution of *Salmonella* serovars amongst the examined samples (*p* > 0.05%), as clarified in Table 2 and Fig. 1.

**Table 2:** The prevalence of *Salmonella* isolated from examined diseased broilers (*n* = 35)

No. of examined birds	Types of organs	*Salmonella* isolates		*S.* Enteritidis *n* = 20		*S*. Typhimurium *n* = 15	
		*n*	%	*n*	%	*n*	%
150	Liver (*n* = 150)	13	8.7	8	5.33	5	3.33
	Spleen (*n* = 150)	8	5.33	4	2.7	4	2.7
	Cloacal swabs (*n* = 150)	14	9.33	8	5.33	6	4
Total	450	35	7.8	20	4.44	15	3.33
Chi square		1.7714		1.6		0.4	
*P* value		0.4124^NS^		0.4493^NS^		0.8187^NS^	

*NS* non-significant

### Antibiogram of the recovered *Salmonella* serovars

The retrieved *S*. Enteritidis serovars were resistant to sulfamethoxazole–trimethoprim (100%), amoxicillin (100%), oxytetracycline (100%), amoxicillin-clavulanic acid and erythromycin (90%), ceftazidime and cefotaxime (85%), gentamycin and neomycin (65%). In contrast, the tested *S*. Enteritidis serovars were sensitive to meropenem (95%) and norfloxacin (85%).

**Fig. 2:**
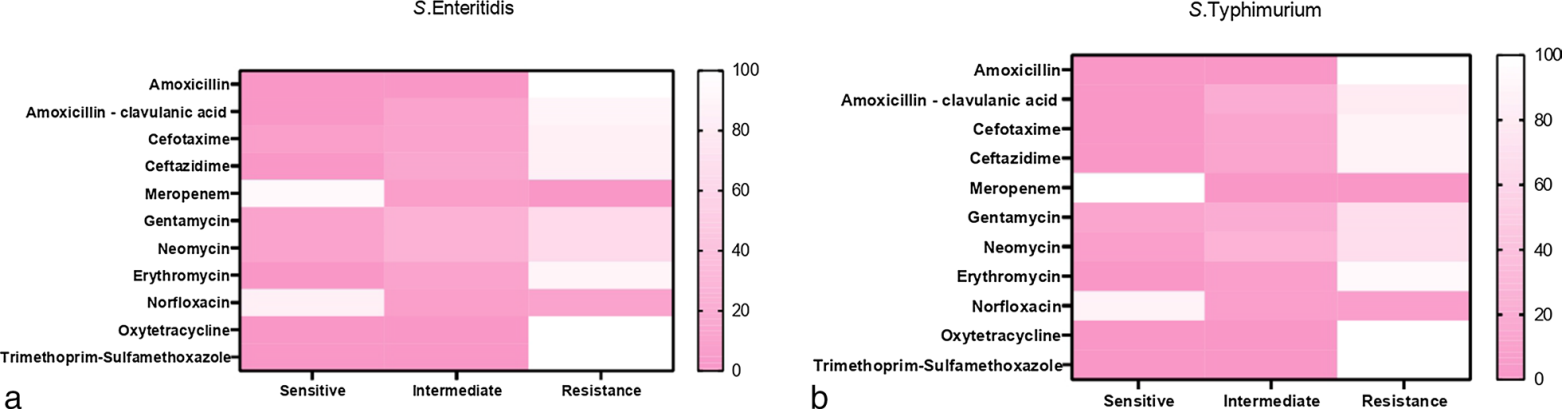
Demonstrates the antimicrobial susceptibility of the obtained **a** *S.* Enteritidis and **b** *S.* Typhimurium serovars isolated from diseased broilers

**Fig. 3:**
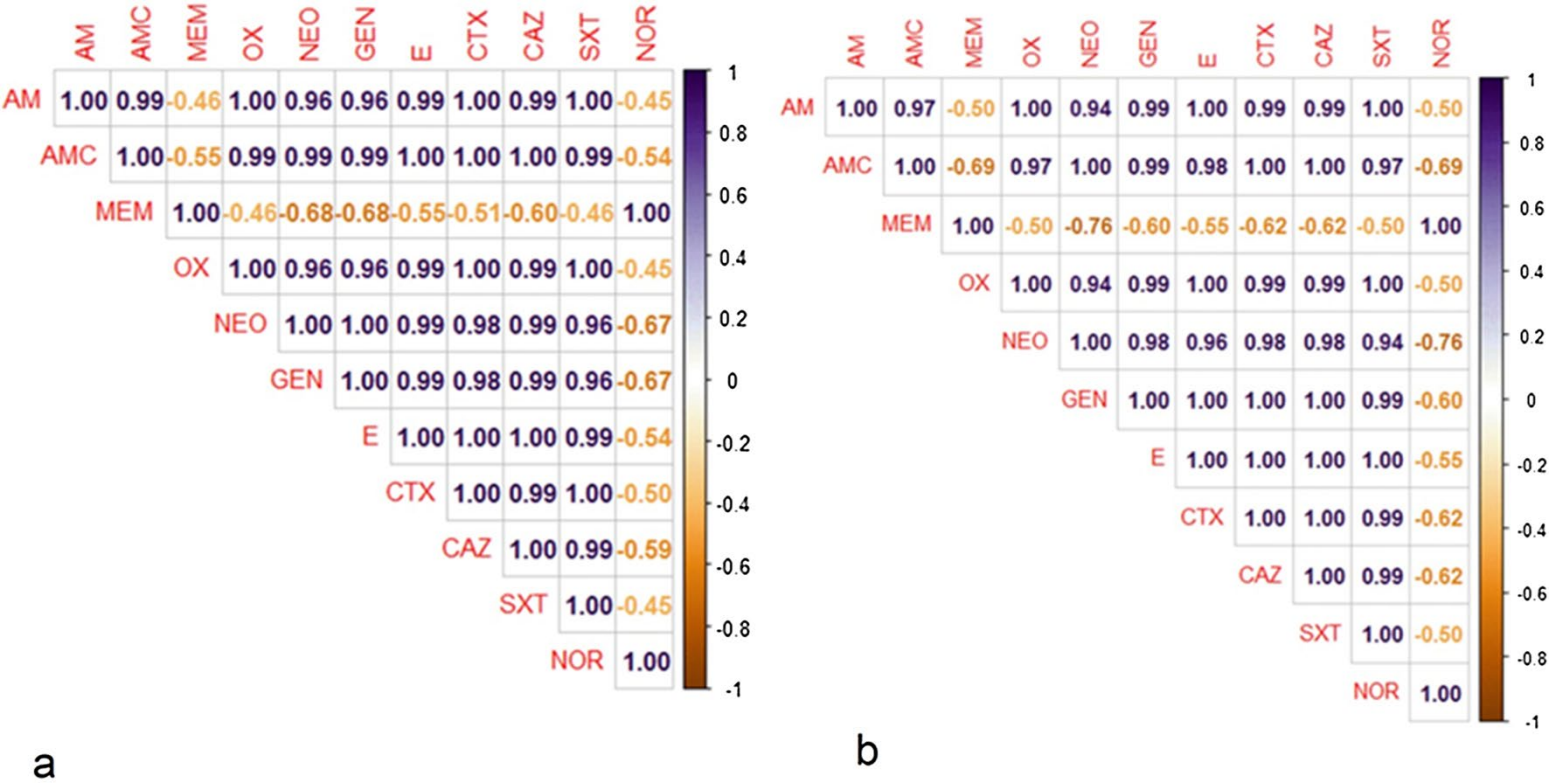
The heatmap establishes the correlation coefficient (r) among various tested antibiotics in the susceptibility testing of the recovered *Salmonella* serovars; **a** *S.* Enteritidis and **b** *S.* Typhimurium

Moreover, the tested *S*. Typhimurium serovars displayed high resistance against sulfamethoxazole-trimethoprim (100%), oxytetracycline (100%), amoxicillin (100%), erythromycin (93.3%), ceftazidime and cefotaxime (86.7% for each), amoxicillin–clavulanic acid (80%), gentamycin and neomycin (66.7% for each). Likewise, tested serovars were sensitive to meropenem (100%) and norfloxacin (86.7%) (Table 3 and Fig. 2). Statistically, the obtained *Salmonella* serovars revealed a marked variation in their susceptibility to various antibiotics (*p* < 0.05). Also, the correlation coefficient between different tested antibiotics was assessed, where strong positive correlations were noticed between; AM, NEO, GEN, OX, SXT, and AMC (r = 0.99); CAZ and CTX(r = 0.99); E and SXT (r = 0.99); E, GEN, and NEO (r = 0.99), CAZ, GEN, and NEO (r = 0.99), CTX, GEN, and NEO (r = 0.98), SXT, GEN, and NEO (r = 0.96), AM, GEN, and NEO (r = 0.96), OX, GEN, and NEO (r = 0.96) (Fig. 3).

**Table 3:** Antibiogram of the isolated *Salmonella* serovars (*n* = 35)

Antimicrobial classes	Interpretation						
	Tested antimicrobial agents	*S.* Enteritidis			*S*. Typhimurium		
		Sensitive *n* (%)	Intermediate *n* (%)	Resistant *n* (%)	Sensitive *n* (%)	Intermediate *n* (%)	Resistant *n* (%)
Penicillins	Amoxicillin	0	0	20(100)	0	0	15 (100)
β-Lactam-β-lactamase-inhibitor-combinations	Amoxicillin–clavulanic acid	0	2 (10)	18 (90)	0	3 (20)	12 (80)
Cephalosporins	Cefotaxime	1(5)	2 (10)	17 (85)	0	2 (13.3)	13 (86.7)
	Ceftazidime	0	3 (15)	17(85)	0	2 (13.3)	13 (86.7)
Carbapenem	Meropenem	19 (95)	1 (5)	1 (5)	15 (100)	0	0
Aminoglycosides	Gentamycin	2 (10)	5 (25)	13 (65)	2 (13.3)	3 (20)	10 (66.7)
	Neomycin	2 (10)	5 (25)	13 (65)	1 (6.7)	4 (26.7)	10 (66.7)
Macrolides	Erythromycin	0	2 (10)	18 (90)	0	1 (6.7)	14 (93.3)
Fluoroquinolones	Norfloxacin	17 (85)	1 (5)	2 (10)	13 (86.7)	1 (6.7)	1 (6.7)
Tetracycline	Oxytetracycline	0	0	20 (100)	0	0	15 (100)
Sulfonamides	Trimethoprim–Sulfamethoxazole	0	0	20 (100)	0	0	15 (100)
Chi square		135.8	17.238	34.709	110.58	14.25	26.864
*P* value		*P* < 0.0001	0.06926	0.00014	*P* < 0.0001	0.1619	0.002736

### The occurrence of virulence determinant and resistance genes in *Salmonella* serovars

PCR proved that the obtained *S.* Enteritidis serovars carried *inv*A, *stn,* *hil*A, and *pef*A virulence genes with a prevalence of 100%, 100%, 90%, and 75%, consecutively. Also, the retrieved *S*. Typhimurium serovars carried *inv*A, *stn,* *hil*A, and *pef*A virulence genes with a prevalence of 100%, 100%, 93.3%, and 73.3%, consecutively.

**Fig. 4:**
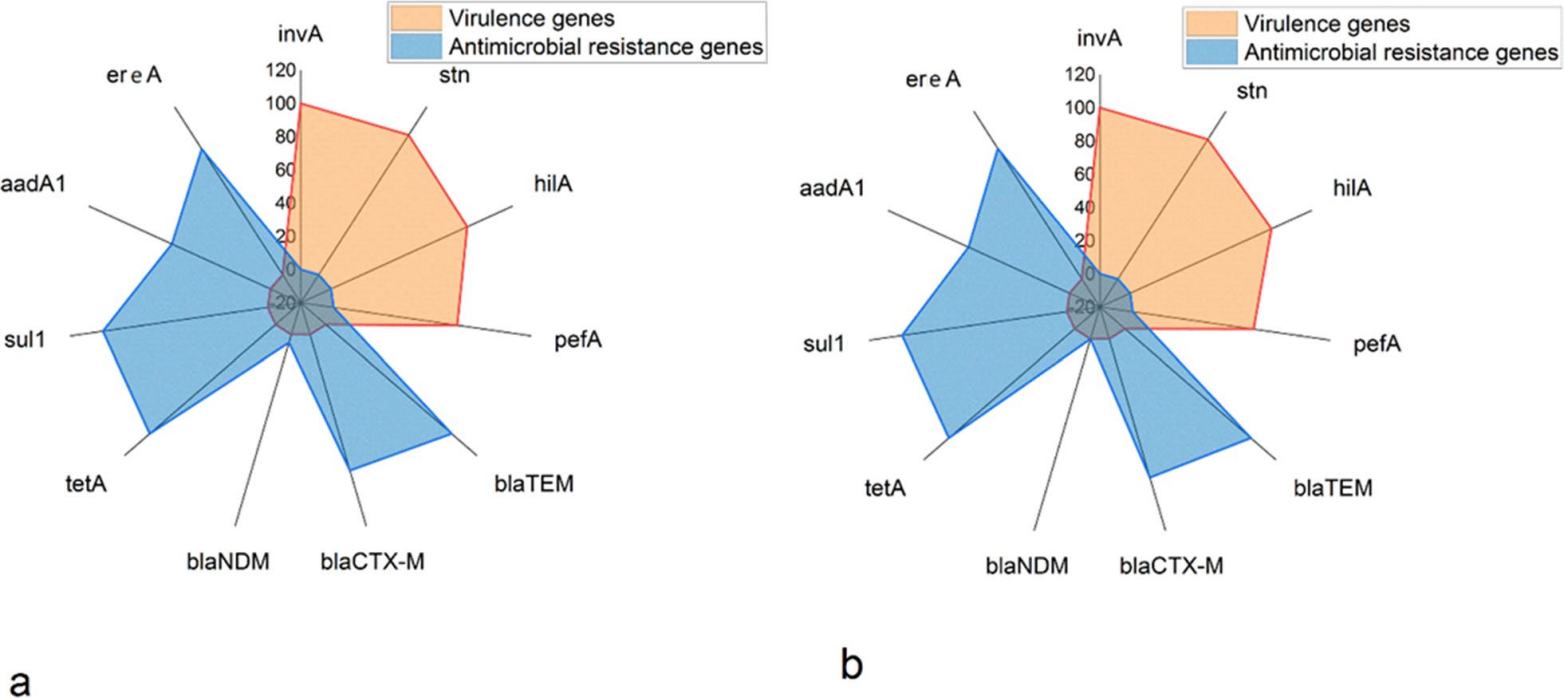
The dispersal of virulence and antimicrobial resistance genes in the tested **a** *S.* Enteritidis and **b** *S.* Typhimurium serovars from diseased broilers

Regarding the occurrence of the antibiotic resistance genes, all the tested *Salmonella* serovars (100%) harbored the *bla*_TEM_, *sul*1, and *tet*A resistance genes. Furthermore, the retrieved *S.* Enteritidis serovars carried *ere*A, *bla*_CTX-M_, *aad*A1, and *bla*_NDM_ resistance genes with a prevalence of 90%, 85%, 65%, and 5%, consecutively. Likewise, the recovered *S.* Typhimurium serovars carried *ere*A, *bla*_CTX-M_, *aad*A1, and *bla*_NDM_ genes with a prevalence of 93.3%, 86.7%, 66.7%, and 0%, consecutively (Table 4 and Fig. 4).

**Table 4:** Dissemination of virulence and resistance genes in the tested *Salmonella* serovars

Types	Genes	*S.* Enteritidis *n* = 20	*S.* Typhimurium *n* = 15
Virulence genes	*inv*A	20 (100%)	15 (100%)
	*stn*	20 (100%)	15 (100%)
	*hil*A	18 (90%)	14 (93.3%)
	*pef*A	15 (75%)	11 (73.3%)
Chi square		0.91781	0.78182
*P* value		0.8211^NS^	0.8538^NS^
Antimicrobial resistance genes	*bla*_TEM_	20 (100%)	15 (100%)
	*bla*_CTX-M_	17 (85%)	13 (86.7%)
	*bla*_NDM_	1 (5%)	0 (0%)
	*tet*A	20 (100%)	15 (100%)
	*sul*1	20 (100%)	15 (100%)
	*aad*A1	13 (65%)	10 (66.7%)
	*ere*A	18 (90%)	14 (93.3%)
Chi square		18.349	15.317
*P* value		0.00541	0.01793

*NS* non-significant

A non-significant difference (*p* > 0.05) was recorded in the dissemination of virulence genes in the tested *Salmonella* serovars. Contrariwise, there was a marked variation (*p* < 0.05) in the distribution of resistance genes between the recovered *Salmonella* serovars.

### Multidrug resistance profiles of the recovered *Salmonella* serovars

Approximately 60% (12/20) of the retrieved *S.* Enteritidis serovars were XDR to seven classes and harbored *sul*1, *bla*_TEM_, *tet*A*,* *bla*_CTX-M_, *ere*A, and *aad*A1genes. Moreover, 25% (5/20) of the obtained *S.* Enteritidis serovars were MDR to six classes and inherited *sul*1, *bla*_TEM_, *tet*A*,* *bla*_CTX-M_, and *ere*A genes. Furthermore, 10% (2/20) of the isolated *S.* Enteritidis serovars were MDR to four classes and inherited *sul*1, *bla*_TEM_, and *tet*A resistance genes. Also, one *S.* Enteritidis serovar (5%) was carbapenem-resistant and XDR to seven different classes and had *sul*1, *bla*_TEM_, *tet*A*,* *bla*_NDM_, *ere*A*,* and *aad*A1 resistance genes.

**Fig. 5:**
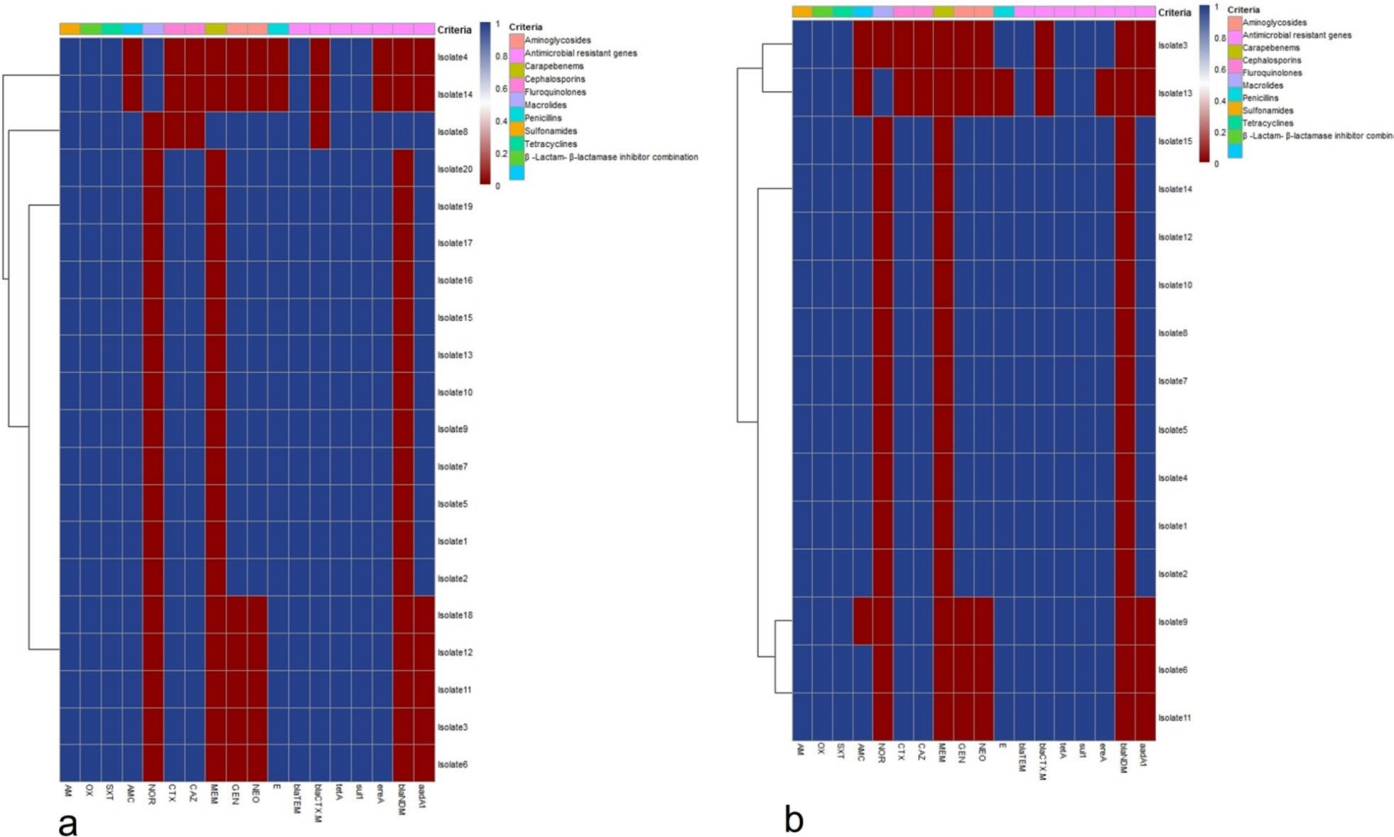
A heatmap shows the occurrence of the antimicrobial resistance phenotypes and antimicrobial resistance genes between the tested **a** *S.* Enteritidis and **b** *S.* Typhimurium serovars. Blue squares indicate the presence of phenotypic and genotypic resistance; red squares indicate the absence of antimicrobial resistance

**Fig. 6:**
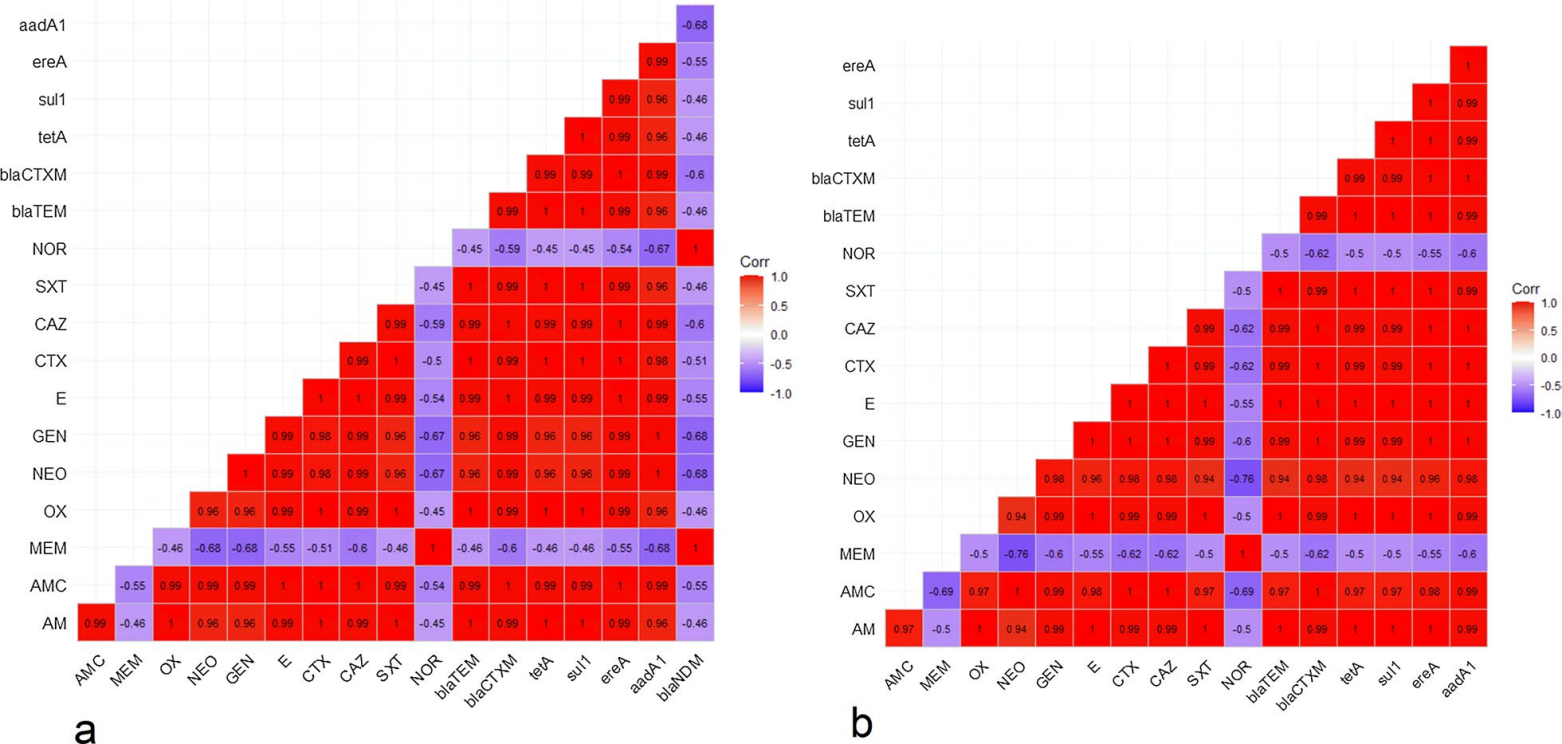
The heatmap reveals the correlation coefficient (r) among tested antibiotics and resistance genes detected in **a** *S.* Enteritidis and **b** *S.* Typhimurium serovars

Likewise, 66.7% (10/15) of the retrieved *S.* Typhimurium serovars were XDR to seven classes and inherited *sul*1, *bla*_TEM_, *tet*A*,* *bla*_CTX-M_, *ere*A, and *aad*A1 genes. Besides, 13.3% (2/15) of the isolated *S.* Typhimurium serovars were MDR to six classes and encoded *sul*1, *bla*_TEM_, *tet*A*,* *bla*_CTX-M_, and *ere*A genes. Also, one *S.* Typhimurium serovar (6.7%) was MDR to five classes and has *sul*1, *bla*_TEM_, *tet*A*,* *bla*_CTX-M_, and *ere*A genes. In addition, one *S.* Typhimurium serovar (6.7%) was MDR to four classes and encoded *sul*1, *bla*_TEM_, *tet*A*,* and *ere*A genes (Table 5 and Fig. 5). Moreover, the MAR index values (0.36–0.82) emphasized various resistance profiles signifying that the tested *S.* Enteritidis and *S.* Typhimurium serovars have emerged from high-risk contamination. The correlation coefficient (r) was determined between the distinguished resistance genes in the isolated *Salmonella* serovars and the tested antibiotics, where positive correlations were noticed between; the *bla*_TEM_ gene and AM (r = 1); *bla*_CTX-M_ and CAZ (r = 1); *sul*1 and STX (r = 1); *ere*A and E (r = 1); *aad*A1, GEN, and NEO (r = 1); *tet*A and OX (r = 1); *bla*_CTX-M_ and CTX (r = 0.99); *bla*_TEM_ and AMC (r = 0.99) (Fig. 6).

**Table 5:** Multi-drug resistance profiles and dissemination of resistance genes among *Salmonella* serovars

No. of serovars	%	Resistance patterns	Phenotypic resistance	Resistance genes	MARI
*S*. Enteritidis (*n* = 20)					
12	60	**XDR**	**9** **Antimicrobial** **agents/7** **classes** SXT, AM, OX, AMC, CTX, CAZ, E, GEN, and NEO	*sul*1,*bla*_TEM_, *tet*A*,* *bla*_CTX-M_, *ere*A, *aad*A1	0.82
5	25	**MDR**	**7** **Antimicrobial** **agents/6** **classes** SXT, AM, OX, E, CTX, CAZ, and AMC	*sul*1, *bla*_TEM_, *tet*A*,* *bla*_CTX-M_, *ere*A	0.64
2	10	**MDR**	**4** **Antimicrobial** **agents/4** **classes** SXT, AM, OX, and NOR	*sul*1, *bla*_TEM_, *tet*A	0.36
1	5	**XDR**	**8** **Antimicrobial** **agents/7** **classes** SXT, AM, OX, E, MEM, GEN, NEO and AMC	*sul*1, *bla*_TEM_, *tet*A*,* *bla*_NDM_, *ere*A*,* *aad*A1	0.72
*S.* Typhimurium (*n* = 15)					
10	66.7	**XDR**	**9** **Antimicrobial** **agents/7** **classes** SXT, AM, OX, AMC, CTX, CAZ, E, GEN, and NEO	*sul*1, *bla*_TEM_, *tet*A*,* *bla*_CTX-M_, *ere*A, *aad*A1	0.82
2	13.3	**MDR**	**7** **Antimicrobial** **agents/6** **classes** SXT, AM, OX, AMC, CTX, CAZ, and E	*sul*1, *bla*_TEM_, *tet*A*,* *bla*_CTX-M_, *ere*A	0.64
1	6.7	**MDR**	**6** **Antimicrobial** **agents/5** **classes** SXT, AM, OX, CTX, CAZ, and E	*sul*1, *bla*_TEM_, *tet*A, *bla*_CTX-M_, *ere*A	0.54
1	6.7	**MDR**	**4** **Antimicrobial** **agents/4** **classes** SXT, AM, OX, and E	*sul*1, *bla*_TEM_, *tet*A, *ere*A	0.36
1	6.7	**MDR**	**4** **Antimicrobial** **agents/4classes** SXT, AM, OX, and NOR	*sul*1, *bla*_TEM_, *tet*A	0.36

## Discussion

Avian salmonellosis is concomitant with high financial crises in poultry farms and severe food-borne illness in man globally (Alam et al. 2020). This work is designed to investigate the occurrence of *S.* Enteritidis and *S.* Typhimurium in diseased broilers, resistance profiles, and PCR detection of virulence and resistance genes.

In the present work, *Salmonella* was isolated from diseased birds suffering from diarrhea and reduced growth performance. The PM examination revealed dehydration, an enlarged congested liver, and an enlarged spleen. Similar results were described by Cocciolo et al. (2020), who recorded that diarrhea, ruffled feathers, anorexia, and pale combs are the predominant clinical signs of *Salmonella* infection in poultry. *Salmonella* infection has an adverse economic impact on the poultry industry due to losses in production, costs of treatment, and mortalities. Moreover, it has public health importance due to the potential transmission to humans, causing foodborne illness (Wajid et al. 2019).

The postmortem inspection of infected birds with *Salmonella* usually exhibited enlarged liver with necrotic foci, enlarged friable spleen, and marked intestinal necrotic foci (Kakooza et al. 2021).

Herein, the retrieved *Salmonella* serovars exposed the typical phenotypic, culture, and biochemical features of *Salmonella* in agreement with Islam et al. (2016). Likewise, the total prevalence of *Salmonella* in the examined diseased broilers was 9.3%, where 35 *Salmonella* isolates (20 *S*. Enteritidis and 15 *S*. Typhimurium) were isolated from 450 samples. Moreover, the highest dissemination of *Salmonella* was noticed in the cloacal swabs. A higher prevalence (35%) of *Salmonella* in broiler chickens was confirmed by Alam et al. (2020). Besides, the highest incidence of *Salmonella* in cloacal swabs was previously reported by Karim et al. (2017). The existence of *Salmonella* in cloacal swabs suggests that bird droppings might represent vehicles for the shedding and transmission of *Salmonella* among chickens (Islam et al. 2016). The emergence of non-typhoidal *Salmonella* serovars in diseased broiler chickens was previously highlighted by Barua et al. (2013) and Alam et al. (2020). The occurrence of *S*. Enteritidis and *S*. Typhimurium infection in broiler chickens suggests the probability of their transmission to human consumers leading to severe food-borne illness (Jajere 2019). Disproportions in the incidence of *Salmonella* could be due to management strategies, biosecurity, sanitary measures, the season of sampling, geographical disparity, environmental stress, species, immune status, and age of the bird (Kumar et al. 2019).

Regarding the antibiogram of the retrieved *Salmonella* serovars, the tested serovars disclosed significant resistance to various classes, for example, tetracyclines, cephalosporins, macrolides, β-Lactams, sulfonamides, and aminoglycosides. These outcomes were nearly consistent with those confirmed by Wajid et al. (2019) and Lapierre et al. (2020). The existence of MDR *Salmonella* serovars is deliberated as a public health concern. The uncontrolled use of antimicrobial agents in the poultry industries, harboring or acquiring *Salmonella* to several resistance genes, resistant plasmids, and integron classes: are the chief causes that recommend the occurrence of these superbugs (Zwe et al. 2018). Hygienic measures and the use of alternatives to antibiotics such as probiotics, prebiotics, and organic acids could reduce the application of antibiotics in poultry farms (Tellez-Isaias et al. 2021).

Concerning the dissemination of virulence determinant genes, the tested *S*. Enteritidis and *S*. Typhimurium serovars usually carried *inv*A and *stn* virulence genes, followed by *hil*A and *pef*A genes. These findings nearly agreed with those confirmed by Ramatla et al. (2020) and Mubarak et al. (2021). The *inv*A gene, the most conserved gene in *Salmonella* species, encodes for a protein, which initiates the invasion of *Salmonella* to the host enterocytes. PCR detection of the *inv*A gene is an accurate and reliable diagnostic tool for the identification of *Salmonella* species such as *S*. Enteritidis and *S*. Typhimurium (Shanmugasamy et al. 2011; Rodriguez et al. 2015). Likewise, *Salmonella* enterotoxin, encoded by the *stn* gene, is presumed the key virulence determinant that is incriminated in diarrhea. The detection of the *stn* gene is valuable for the diagnosis of *Salmonella* infection as it is unique to the *Salmonella* species (Lee et al. 2009). Moreover, the *hil*A gene codes the OmpR/ToxR family transcriptional regulator, which triggers the expression of invasion genes due to external stimulators (Thung et al. 2018). Furthermore, the *pef*A gene is responsible for the adhesion of the pathogen to the host enterocytes (Webber et al. 2019).

With reference to the phenotypic resistance profiles and the dissemination of resistance genes, most of the obtained *S*. Enteritidis and *S.* Typhimurium serovars were XDR to 7 classes possessing *sul*1, *bla*_TEM_, *tet*A*,* *bla*_CTX-M_, *ere*A, and *aad*A1 genes. Multiple-drug resistance is one of the foremost risks to public health worldwide. It was developed attributable to the inappropriate application of antibiotics in poultry farms and the health sector, and the transmission of resistance genes among bacterial pathogens, the presence of resistant plasmids and integrons classes (Soler and Forterre 2020; Rodríguez-Beltrán et al. 2021). The resistance to sulfonamides, penicillins, tetracyclines, and cephalosporins is mainly attributed to the presence of *sul*1, *bla*_TEM_, *tet*A*,* and *bla*_CTX-M_ resistance genes, respectively (McMillan et al. 2019). Likewise, the aminoglycosides resistance occurred via the enzymatic modification pathway enhanced by adenylyltransferase (coded by the *aad*A1 gene) with subsequent inactivation of aminoglycosides antibiotics (Ramirez and Tolmasky 2010). Besides, the resistance of *Salmonella* serovars to erythromycin is commonly enhanced by erythromycin esterase (encoded by the *ere*A gene) (Katiyar et al. 2020). Worryingly, in the present study, one *S.* Enteritidis serovar is carbapenem-resistant carrying the *bla*_NDM_ gene reflecting a public health threat. A previous investigation (Parvin et al. 2020) revealed the occurrence of carbapenem-resistant *Salmonella* strains carrying the *bla*_NDM-1_ in chicken meat in Bangladesh as a first report.

Concisely, this study underscored the re-emergence of XDR *S*. Enteritidis and *S*. Typhimurium serovars in diseased broilers. The retrieved *S*. Enteritidis and *S*. Typhimurium serovars usually carried the *inv*A and *stn* virulence genes, followed by *hil*A and *pef*A genes. Most of the obtained *S*. Enteritidis and *S.* Typhimurium serovars were XDR to several classes and inherited *sul*1, *bla*_TEM_, *tet*A*,* *bla*_CTX-M_, *ere*A, and *aad*A1 genes. Meropenem and norfloxacin exposed a hopeful antimicrobial activity to XDR *S*. Typhimurium and *S.* Enteritidis in diseased broilers. The combination of conventional and molecular assays is a dependable implement for monitoring salmonellosis in poultry. Threateningly, the re-emergence of XDR *Salmonella* serovars launches a public health threat. As a result, it inspires the predictable application of antibiotic susceptibility and the correct application of antibiotics in the poultry industry and health sector.

## Data Availability

Not applicable.
